# Negative Work Attitudes and Task Performance: Mediating Role of Knowledge Hiding and Moderating Role of Servant Leadership

**DOI:** 10.3389/fpsyg.2022.963696

**Published:** 2022-07-22

**Authors:** Zailan Tian, Chao Tang, Fouzia Akram, Muhammad Latif Khan, Muhammad Asif Chuadhry

**Affiliations:** ^1^School of Management, Guangdong Polytechnic Normal University, Guangzhou, China; ^2^Department of Business Administration, University of Prince Mugrin, Madina, Saudi Arabia; ^3^Global College of Engineering and Technology, Muscat, Oman; ^4^Department of Management Science, Shifa Tameer-e-Millat University, Islamabad, Pakistan

**Keywords:** servant leadership, job insecurity, knowledge hiding, employee cynicism, role stress and task performance

## Abstract

The COVID-19 pandemic has caused a global crisis that particularly hit employment globally. Due to the economic crisis, many small businesses attempted to minimise their expenses by either closing or downsizing. During such organisational situations, the employees face negative workplace attitudes that lead to knowledge hiding and affect team performance. This study examines negative attitudes and their effect on team performance. Further, this study examines the mediating effect of knowledge hiding and moderating the role of servant leadership. Through a multi-time data collection approach, the authors obtained 363 responses from the education sector in China during the COVID-19 pandemic. PROCESS Hayes model 1 and 4 were used for mediation and moderation analysis. Results show that job insecurity, cynicism, and role stress are significant forces behind knowledge-hiding behaviour. Furthermore, the knowledge hiding behaviour adversely affects task performance. Servant leadership shows a buffering effect on knowledge hiding behaviour caused by negative workplace attitudes. This is one of the first studies in the South Asian environment to examine the association between employees’ negative attitudes and task performance using knowledge hiding as a mediator and servant leadership as a moderator in the COVID-19 scenario. Lastly, the paper concludes with a consideration of its theoretical, practical implication and future direction.

## Introduction

Employees’ attitudes have been adversely impacted due to the present COVID-19 situation (Malik and Sanders, 2021). The COVID-19 pandemic is considered one of the most devastating tragedies in the history of human history, spreading over the globe at an alarming rate and never seeming to end (Djalante et al., 2020). While dealing with the pandemic, organisations start downsizing to lower their expenses (Malik, 2013; Ngoc Su et al., 2021). Organisational instability leads the enthusiastic staff to suffer from role stress, employee cynicism, and insecurity in the workplace, all of which lead to purposeful attempts to hide knowledge (Chen M. et al., 2021; Zada et al., 2021). Employees are reluctant to share knowledge and show less enthusiasm because they are concerned about not having a purpose in their jobs (Welbourne Eleazar and Park, 2021). Employees are more likely to keep their knowledge to themselves to preserve a competitive advantage in an environment where job security is not guaranteed (Zhang et al., 2020; Ali et al., 2021a). Individuals who work in such an environment may become disillusioned about their workplace, which may have a variety of adverse outcomes, i.e., knowledge hiding, which ultimately affects team performance (Barry and Wilkinson, 2021).

Knowledge hiding behaviour has several significant negative consequences (Butt and Ahmad, 2020). The worst incident documented in 2018 was the productivity loss of enterprises in the United States due to knowledge hiding, which might have cost up to US$ 47 million (Panopto, 2018). According to Panopto (2018), American employees waste around 5.3 h per week waiting to get existing knowledge or information from their co-employees. The productivity and growth of businesses suffer a lot, which reduces the absence of harmony among employees, and the disobedience of employees toward organisation regulations (Barry and Wilkinson, 2021; Chen D. et al., 2021). Despite employers’ encouraging attempts to encourage knowledge sharing and employee voice-over employee behaviour, many people hide knowledge and deliberately withhold vital knowledge from team mates (Nguyen et al., 2022). Employees willfully hold or hide knowledge when their colleagues request it (Connelly et al., 2012). In a workplace where employees are expected to reciprocate social exchanges, less knowledge is hidden (Serenko and Bontis, 2016). Situations like COVID-19’s significant loss of economy encouraged employees to hide their knowledge to protect their employment (Malik and Sanders, 2021; Zada et al., 2022a). According to the conservation of resource theory (COR), the employees might see the knowledge hiding conduct as self-serving resource preservation (Hobfoll, 1989). They are naturally inclined to safeguard the limited available resources, especially during times of crisis (Anand et al., 2020).

In times of crisis, servant leadership significantly impacts overcoming challenges and reducing organisation problems (Zhang et al., 2021). Serving leadership has received much attention because of its connection to employees’ responses (Schmid et al., 2018; Siyal et al., 2021b). Tuan (2016) found that servant leadership benefits employees’ willingness to share knowledge. Servant leader stimulates and motivates employees to take on challenges, accept adjustments, and inspire them to innovate in their roles as members of the organisation. Despite its sound effects, little study has been identified on how servant leadership affects knowledge hiding practices, including actively delaying the precise knowledge needed by colleagues. Employee task performance might be affected if the practice of withholding knowledge continues, so the research mentioned above research gap must be filled. No one has looked at how servant leadership influences the antecedents of knowledge hiding attitude, yet we have read about it in the literature (Saeed, 2018; Siyal and Peng, 2018; Farid et al., 2021).

**FIGURE 1:**
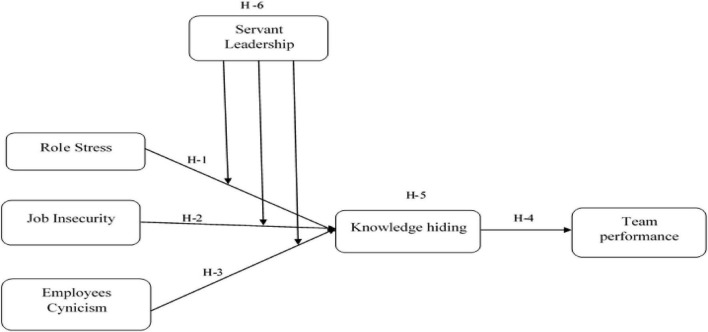
Conceptual model.

According to the Figure 1 this study is focussed on two key objectives: (1) identifying the antecedents and consequences of purposeful knowledge hiding conduct due to negative work attitudes and (2) examining how servant leadership influences knowledge hiding behaviour and its effect on task performance. Following these aims, this study intends to give a combined new understanding of negative employee attitudes that lead to knowledge hiding in a high crisis. Examining the impacts and causes of hiding knowledge at the worldwide crisis that has affected almost every organisational setup still contributes to the knowledge hiding literature. In addition, this research adds to our understanding of the underlying processes of interaction between antecedents of knowledge hiding behaviour and servant leadership. It provides evidence of the moderating influence of servant leadership. The findings of this study will be helpful to researchers studying servant leadership roles in crisis management and resource conservation.

## Theory Development

Conservation of resources theory (COR) supports the study model. The COR theory explains how employees’ restore their valuable resources from being lost (Hobfoll, 1989), after facing negative attitudes. Such negative attitudes affect team performance. Employees will do all their measures to secure their valuable resources if they identify any threat (Hobfoll, 1989; Simha et al., 2014). Cynicism, role stress, and job insecurity are possible causes, often followed by knowledge hiding and affecting team performance. Employees who are under such strain may hide knowledge to secure resources (Naus et al., 2007). Previous study suggests that COR theory has thoroughly explored and focussed on fundamental factors. Work/family stress (Ruiz-Palomino et al., 2022), general stress (Halbesleben, 2006), and burnout are examined (Halbesleben et al., 2009). This study extends the COR research by finding and adding additional factors such as personality traits that may be precursors to knowledge hiding behaviour.

In this research, we studied that knowledge hiding behaviour, which results from role stress, job insecurity, and cynicism, has received little attention in earlier literature. Employees do not complete their tasks because of the stress of working under job insecurity, cynicism, work deadlines, and fear of losing their jobs (Cole et al., 2012). According to the COR theory Hobfoll (1989), employees prefer to retain their resources to avoid future losses. While under stress, cynicism, and job insecurity, employees are more likely to engage in knowledge-hiding activities (Ahmad et al., 2021a). When information is kept secret and resources are preserved, employees have a greater sense of safety and psychological well-being (Hernaus et al., 2018). Several important factors, including role stress, job uncertainty, and cynicism, were not addressed while examining the underlying reasons for the prior work’s knowledge hiding attitude. In addition, these data are critical for gaining a deeper understanding of employees’ behaviour with task performance. Parris and Peachey (2013) claim that employees’ reactions to resource loss are influenced by servant leadership in an organisational crisis. According to prior studies, such as (Tripathi et al., 2021), servant leadership acts as an accelerator in terms of encouraging people to share expertise and preventing them from knowledge hiding.

### Hypotheses Development

#### Role Stress and Knowledge Hiding

Multitasking conflicts and multi-role expectations cause role stress (Zhao and Jiang, 2021; Khan et al., 2022c). “Role stress” refers to unstable situations in which people feel uncertain, role inconsistency, or overloaded expectations. It includes a behavioural character that includes three main aspects, i.e., the ambiguity of the spirit, conflicting behaviour, and overload (Kahn, 1990). Stress-related to one’s job is a regular occurrence in the workplace. When an organisation faces a crisis, employees tend to gravitate more strongly toward stress-related behaviours (De Dreu et al., 2004). Role stress is associated with the imbalance in interpersonal interactions. We postulate that the hiding of knowledge is influenced by role stress. According to the COR theory by Hobfoll (1989), when employees are affected by role stress, they engage in knowledge hiding behaviour. This notion reduces the reciprocal reliance on the organisation’s personnel. Role stress is a term used to describe the negative relationship between an organisation and its employees. Employees become doubtful about their work and cannot feel the satisfaction of task completion. In the COR theory Hobfoll (1989), behaviour such as knowledge hiding is caused by negative social connections, such as the stress of role-playing, which might have unfavourable repercussions. Retaliation inclinations are heightened when employees are stressed from their jobs (Schulz-Hardt et al., 2002). Employees may react adversely and hide knowledge (Anand et al., 2020; De Clercq et al., 2021). As a result, it became clear that role stress is the driving force behind people’s tendency to hide knowledge. Workers who assume the role stress is likely to generate fewer mutually satisfying results is a similar notion (Schulz-Hardt et al., 2002). Workplace tensions are often exacerbated by the clash of opposing viewpoints and ideas (Siyal et al., 2021a). Workplace stress may be caused by conflict and hatred amongst colleagues. Role stress makes employees more likely to hide their abilities if they feel disrespected or unjustly treated (Anand et al., 2020). Due to increasing role stress, it is projected that employees would keep their expertise hidden from colleagues. Based on this, the following hypothesis could be derived:

**H1**: There is a positive relationship between role stress and knowledge hiding

#### Job Insecurity and Knowledge Hiding

The term “job insecurity” refers to the fear that one’s employment may be terminated at any moment (Russo and Terraneo, 2020). According to COR theory, employees’ fear of losing their jobs drives them to hide their knowledge (Jha and Varkkey, 2018). Knowledge is seen as a source of power and a symbol of employment stability (Ali et al., 2021b; Saeed et al., 2022). Job-insecure employees believe that not sharing their expertise is a way to protect their competitive advantage in the workplace (Butt and Ahmad, 2020). Sharing their knowledge, experience, and expertise with others helps them to be replaced, according to these employees (Issac et al., 2020). The high level of job insecurity among employees makes them vulnerable to knowledge hiding (Issac and Baral, 2018). Employees assume that sharing their experience, knowledge, or talent will put them at risk of being replaced by their employer (Issac and Baral, 2018). Employment insecurity leads employees to hide knowledge from their colleagues (Ali et al., 2021a). COR theory stated that employees concerned about losing their jobs tend to be less cooperative than those who aren’t (Cheng et al., 2005). Job security is one of three critical motivators for enhancing employee engagement, offering aid, sharing experience, and enhancing job performance (Senol, 2011; Zhuang et al., 2021). Job insecurity and a high turnover in the workforce are the most typical reasons for knowledge hiding. Uncertainty at work is a significant contributor to undesirable behaviours such as knowledge hiding, contemplation of leaving one’s position, and a decline in one’s ability to be creative in one’s work. So, we hypothesised:

**H2:** There is a positive relation between job insecurity and knowledge hiding

#### Employee Cynicism and Knowledge Hiding

Employee reactions to organisational change have gotten much attention in the last decade (Kim and Kim, 2020; Hu et al., 2021). Furthermore, employees’ positive role is critical to an organisation’s success (Mao et al., 2021). It is essential to manage employee cynicism in the context of an organisation crisis because it hinders organisation performance (Sguera et al., 2021). According to COR theory, employees who are cynics doubt their relevance in organisations and feel there aren’t valuable resource for an organisation and ultimately choose hide knowledge (Naeem, 2020). Employees who become cynic due to workplace stress may hide knowledge (He et al., 2021; Khan et al., 2021a; Zada et al., 2022b).

Furthermore, cynicism involves negative conduct on the part of employees about their position in the organisation (Megeirhi et al., 2020). As a result, the worker is reluctant to collaborate with others and demonstrates a reluctance to share vital expertise. In some instances, cynicism is sometimes related to unethical work (Ahmad et al., 2021b). Cynicism may be responsible for a person’s unwillingness to offer knowledge that others need (Aljawarneh and Atan, 2018). Cynicism strongly supports knowledge hiding since it prevents employees from focusing on appropriate work habits (Jiang et al., 2019; Khan et al., 2021b). Cynicism is too closely associated with the experience of having a poor degree of job achievement (Bang and Reio, 2017). Such employees are dissatisfied with their jobs and cynical about their value to the organisation (Cartwright and Holmes, 2006). Many negative factors are linked to cynicism, such as a lack of enthusiasm for work, leading to unpleasant emotions (Cole et al., 2006). Employees have a cynical attitude due to organisational crisis, and engage in knowledge hiding (Reichers et al., 1997). In other words, cynic employees seem more focussed on not interacting with their co-employees to avoid learning about hidden talents, ideas, and knowledge. Cynicism among employees breeds distrust (Stanley et al., 2005). Employees can’t work together if they don’t have any social connections. In this way, we may make the following hypothesis:

**H3**: There is positive relation between employee cynicism and knowledge hiding

### Knowledge Hiding and Task Performance

Knowledge hiding effect task performance in three ways (Cooke et al., 2019). First, the culture of knowledge sharing is directly associated with better job task performance (Xiao and Cooke, 2019). Second, employees with a negative attitude engaged in knowledge hiding create a unpleasant atmosphere. In such atmosphere employees cannot trust coworkers, and not prepared to seek or provide assistance to others whenever requested (Xiao and Cooke, 2019). This type of behaviour decreases overall task performance. Third, professional jealousy is another cause of knowledge hiding, that effect task performance (Xiao and Cooke, 2019).

In many organisations, individuals obligated to share knowledge to enhance employee’s task performance. Knowledge hiding becomes an obstacle in the systematic process of transferring, dispersing, and distributing knowledge (Chen et al., 2011). Employees’ job task performance may be improved by encouraging knowledge sharing in a learning culture. The goal of the knowledge-sharing is to enhance job related knowledge to increase task performance (Huang et al., 2021) Consequently, employees’ job performance is often reduced due to knowledge hiding, mainly for three reasons: lowered problem-solving abilities, reduced decision-making abilities, and a lack of creative imagination (Debus and Unger, 2017). It is more challenging to use existing knowledge to create new products and services when employees withhold knowledge due to a culture of knowledge hiding (Peng, 2013; Serenko and Bontis, 2016). Employees’ ability to perform their jobs effectively, knowledge hiding is one of the main causes to reduce its efficiency (Wang and Noe, 2010; Ma and Bennett, 2021). Employees unable to cooperate across organisational to produce innovative ideas due to knowledge hiding, making it impossible for them to acquire task-related knowledge (Chen et al., 2011). So, we can hypothesise that:

**H4:** There is a negative association between knowledge hiding and task performance

### Mediation Effect of Knowledge Hiding

Organisational bad performance is the main reason behind employee cynicism, stress, and job insecurity (Debus and Unger, 2017). At times of crisis, an organisation’s employees lose their trust and confidence because they lack the necessary resources to do their tasks (Cartwright and Holmes, 2006). Whenever there is instability in the organisation due to performance, employees begin to worry about their employment, which causes stress and makes them cynic (Delfino and van der Kolk, 2021). When individuals associated with an organisation begin to work just for their benefit, other employees think they have the least trust in their peers (Cole et al., 2012). According to the COR theory developed by Hobfoll (1989), role stress, employee cynicism, and job insecurity are all factors that cause employees to engage in knowledge hiding behaviour to safeguard their limited resources. They hesitate to share their experience and valuable abilities with their colleagues to avoid further losses and assure their job security. The process of knowledge sharing seems to have come to a halt due to employees withholding information and being unable to learn from one another. Due to instability in the organisation performance, employees become unable to share information and have a detrimental impact on productivity and task performance (Wang and Noe, 2010). To sum up, we may conclude that role stress, job insecurity, and employee cynicism leads to knowledge hiding, and when there is a culture of knowledge hiding, overall task performance affected in a negative way.

**H5:** Knowledge hiding is mediates the link between (I) role stress, (II) job insecurity, (III) employee cynicism and task performance.

### Moderating Role of Leadership

Servant leadership encourages, motivates, inspires and helps employees develop a knowledge-sharing culture. Such kind of knowledge culture effect employee’s task performance (de Waal and Sivro, 2012; Reslan et al., 2021). Servant leadership encourages employees to perform and attain organisation objectives (Brière et al., 2021). Under servant leadership, when employees face challenges, servant leadership help and courage them to seeks different ways get out from all challenges (Yoshida et al., 2014). Previous studies have shown that servant leaders’ behaviour lowers the organisation’s knowledge hiding culture (Bilal et al., 2020). Servant leadership motivates the workforce to reach common goals, reward employees to contribute to the organisation, and promote good attitudes toward knowledge-based behaviours to lessen the workplace’s stress, cynicism and job insecurity (Franco and Antunes, 2020). Servant leaders are known to assist their employees in developing organisational citizenship behaviour (OCB) to reduce the likelihood of hiding knowledge from their colleagues.

Servant leadership has a moderating effect to reduce role stress, job insecurity, and employee cynicism that furthers keep them away from knowledge hiding. Servant leadership helps organisation in times of crisis by increasing employees’ motivation, morale, and performance via a series of interconnected positive actions (Hoang et al., 2021). Research shows four different deriving factors behind such a potential servant leadership role. First, servant leadership give employees a platform where they may flourish, share knowledge and play effective role in the task performance. Second, servant leadership intellectually stimulates the employees and inspires them to embrace work challenges. Third, servant leadership provides favorable conditions for knowledge sharing by increasing mutual trust and interaction among colleagues, motivating them to help each other (Reslan et al., 2021; Zhang et al., 2021). Fourth, it underlines the importance of employees’ contributions toward their task performance (Chughtai, 2019). Accordingly, we hypothesised that:

**H6:** Servant leadership moderates the impact of (I) role stress, (II) job insecurity, and (III) employee cynicism on knowledge hiding

## Materials and Methods

The present research is quantitative and self-reported, collected at three different intervals (After 30-days). This was done to limit the likelihood of common method biases (Podsakoff et al., 2012). In view of Podsakoff et al. (2012), the amount of time spent gathering data ought to be too lengthy or unduly short. When there is excessive time between observations, a number of confounding variables may disguise the presence of a connection between the variables (Babalola et al., 2017). However, memory effects may lead to an unnaturally exaggerated relationship between the variables if the time lag between occurrences is too short (Babalola et al., 2017). An approach based on convenience sampling technique was used in this study because of the unavailability to get official statistics on the total number of Chinese educational sector employees.

### Data Collection and Pretesting

Employees of educational institutes in china were asked to complete self-reported questionnaires. Due to their busy schedules, employees are difficult to contact without a personal recommendation. The authors visited each organisation to collect data. Before data collection, the management of organisations gave formal permission to distribute surveys. The authors approached the employees during their office timing and told them about the purpose of the study. Data were collected only from those employees who volunteered to participate in the study. The researchers visited them at time 1 to collect data for the independent variables, namely role stress, job insecurity, employee cynicism, and demographics. After a gap of 30 days, the researchers visited the organisations to collect data on servant leadership and knowledge hiding from those employees who had already provided data for 1st time. After waiting for another 30 days, the researchers personally visited the organisations to collect time three on task performance.

In the top right corner of the questionnaire, all respondents were asked to generate a unique identification number by inputting their first name and last name, followed by their birth date, in the area given. Three-time lags were considered while compiling this survey of the employees’ responses. The authors ensured that all respondents submitted this identification number throughout each of the three-time lags. This one-of-a-kind was identifying key assisted in combining the replies provided by each employee across a three-time lag. To conduct the data analysis, it was decided to utilise just those employees’ data who completed the survey three times. The questionnaire was divided into two sections. The first portion consisted of a cover letter explaining the research goal and the process used to generate the unique I.D and the second part consisted of a questionnaire. When time 1 concluded, only 443 completed questionnaires out of 473 respondents. After 30 days, the same 443 respondents were contacted again to gather data for the study’s second phase. The second time, only 398 respondents returned the questionnaires. After 30 days, the same 398 respondents were contacted again to acquire replies for time three. After time three, a total of 363 completed replies had been received, which were utilised for data analysis. The response rate for this survey was 93.31 percent.

### Measures

#### Roll Stress

To measure the role stress, a 4-item scale was used (Cohen et al., 1983). For instance, “I am constantly drawn into conditions in which there are conflicting requirements.” The value of Cronbach’s coefficient α was calculated as 0.892.

#### Job Insecurity

The seven-item scale developed by Vander Elst et al. (2014) was utilised for measuring job insecurity. A sample of it is: “Chances are I will soon lose my job.” For job insecurity, Cronbach’s alpha coefficient comes out to be 0.97.

#### Cynicism

For the measurement of cynicism, adapted from the study of Schaufeli et al. (2002). An example of it is, “Chances are I will soon lose my job.” Calculated for cynicism cronbach’s alpha coefficient was 0.90.

#### Knowledge Hiding

Knowledge hiding was measured by using four-item scale was adapted from (Peng, 2012). A sample of it is “I do not want to transfer personal knowledge and experience to others”. For knowledge hiding value of Cronbach’s alpha coefficient was 0.89.

#### Task Performance

To measure the task performance of the employee with seven scales developed by Williams and Anderson (1991) were used having a reliability coefficient of *a* = 0.97. Sample items were “This employee adequately completes assigned duties” and “This employee meets formal performance requirements of the job.” The scale was found to be valid and reliable to be used for measuring the employee’s task performance.

#### Servant Leadership

To measure servant leadership, a 7-item scale was adapted from Liden et al. (2015). Items with good to excellent Cronbach alpha values that is α = 0.95. The items included scrutinised the leadership effectiveness from the employee’s perspective, such as “My supervisor makes my career development a priority.”

### Demographics

Demographics show that 36.6% of respondents were female, whereas 63.4% were male. 6.6% of respondents were between 25 and 30 years of age, 57.0% respondents were between 31 and 35 years of age, 19.8% were between 36 and 40 years of age, whereas the remaining 16.5% of respondents were more than 40 years of age. 4.1% of the employees had an HSSC, whereas the 11.6% had completed a bachelors’ degree and master’s and MS/PhD were 78.8 and 5.5%, respectively. Minimum, one year of experience in the organisation, was set as an inclusion criterion. 45.7% of employees had 1 to 5 years of working experience, 4.3% of the respondents had more than 16 years of total working experience.

## Results

### Control Variables

Respondent’s age, gender and educational level were kept under control. These has been found to effect task performance of employees. Gender is dummy coded (one = male; two = female). The respondents’ educational levels are evaluated using four different criteria in the survey (one = HSSC; two = Bachelor’s; three = Master degree; four = MS/Phil and PhD) (Kim et al., 2017).

### Common Method Variance

We performed Harman’s one-factor test (Podsakoff, 2003) before investigating the hypotheses to rule out any interferences owing to common method variation (Podsakoff, 2003). This test indicated that a common method factor explained 21.3 per cent of the total variance in the results. Based on this assumption, we believe that our results are not significantly impacted by considerable common method variation (21.3% < 50%).

### Mean, Standard Deviation, Pearson Bivariate Correlation, and Cronbach Alpha Reliabilities

Table 1 reports the study variables’ means, standard deviations, and correlations. Consistent with our theoretical expectations, the zero-order correlations for role stress, job insecurity, employee cynicism, knowledge hiding, servant leadership and task performance were all in the expected direction, with the strongest correlation between job insecurity and employee cynisism (*r* = 0.475, *p* < 0.01). See Table 1 for further details.

**TABLE 1:** Mean, SD, correlations and reliability.

Variables	Mean	SD	1	2	3	4	5	6	7	8	9	10
1. Gender	1.366	0.4824										
2. Age	2.462	0.8446	–0.031									
3. Experience	1.727	0.8005	0.166	0.03								
4. Education	2.856	0.5625	0.001	0.04	–0.038							
5. Servant leadership	3.762	0.7058	–0.005	0.02	0.069	0.065	**(0.81)**					
6. Role stress	3.801	0.7114	0.055	0.02	0.037	–0.009	−0.451**	**(0.77)**				
7. Job insecurity	3.887	0.7912	0.064	0.06	0.037	0.099	−0.532**	0.264**	**(0.88)**			
8. Employee cynicism	3.752	0.7039	0.076	0.07	0.025	0.253**	−0.557**	0.342**	0.475**	**(0.78)**		
9. Knowledge hiding	2.965	0.7989	–0.019	–0.03	–0.018	0.018	−0.115*	0.228**	0.169**	0.127*	**(0.82)**	
10. Task performance	3.685	0.6697	0.025	0.04	0.069	0.071	0.429**	−0.356**	−0.299**	−0.410*	−0.497*	**(0.86)**

**Correlation is significant at the 0.01 level (2-tailed).

*Correlation is significant at the 0.05 level (2-tailed).

*The bold values indicated the cronbach’s alpha.*

### Confirmatory Factor Analysis

Tor confirms the convergent and discriminant validity of study variables we run confirmatory factors. The Maximum Likelihood (ML) method was used to estimate parameters in CFA models. Literature suggests using the ML method for social and behavioral sciences studies, which involves Likert scale items (Li, 2016). Convergent validity was tested by examining each item’s estimated scores, also known as factor loadings. For this study, the factor loading values show the lowest is 0.71, and the highest is 0.88. Each factor fully loads on its associated latent variable, showing convergent validity. According to research studies, convergent validity is proven when the factor loadings are equal to or greater than 0.3 (Hu and Bentler, 1999; Heene et al., 2011). Additionally, the discriminant validity was assessed using the Maximum Likelihood approach for estimating the parameters of CFA models. This included checking the values of model fit indices such as CFI, TLI, and RMSEA. The results of the four-factor model’s (See Table 2) were tested with a one-factor model in AMOS. Model fitness was shown good by a four-factor model with statistics X^2^ = 3174, df = 1237, TLI = 0.90, CFI = 0.91, RMSEA = 0.03, SRMR = 0.05, as compared to the one-factor model. Research studies show that proposed model will be good if *p* > 0.05 for χ^2^, CFI, IFI, and TLI > 0.80 and RMSEA < 0.08 (Yu et al., 2002). Overall, the confirmatory factor analysis revealed a high index of model fit.

**TABLE 2:** Results of the confirmatory factor analysis (*N* = 363).

Model	X^2^	df	TLI	CFI	RMSEA	SRMR
Hypothesised four-factor model	3174	1237	0.90	0.91	0.03	0.04
Three-factor model	3690	1372	0.74	0.63	0.08	0.06
Two-factor model	3756	1577	0.53	0.44	0.13	0.17
One-factor model: SL, KH, RS, JI, EC and TP	4147	1765	0.42	0.34	0.17	0.19

*X^2^, normal-theory weighted least-squares Chi square. TLI is the Tucker–Lewis fit index, CFI, the comparative fit index, RMSEA, the root-mean square error of approximation, and SRMR, the standardised root-mean-square residual. SL, servant leadership; KH, knowledge hiding; RS, role stress; JI, job insecurity; EC, employee cynicism; TP, task performance.*

### Results for Direct and Indirect Effect

Table 3 states that the first hypothesis of the current study states that role stress is positively linked to knowledge hiding (β = 0.256, *p* > 0.000), leading to the acceptance of H1. Hypothesis 2 proposed that job insecurity is significantly associated with knowledge hiding (β = 0.271, *p* > 0.000), supporting H2. Hypothesis 3 stated that employee cynicism is positively related to knowledge hiding (β = 0.345, *p* > 0.000), supporting hypothesis 3. Hypothesis 4 stated a negative relation between knowledge hiding and task performance (β = –0.053, *p* > 0.06). The bootstraps result for indirect effect at a 95% confidence interval showed that knowledge hiding mediates the relationship between role stress and task performance (β = 0.0835), CI [0.0472, 0.1219]. Further, knowledge hiding mediates the relationship between job insecurity and task performance (β = 0.0689), CI [0.264,0.1105], and also mediates the relationship between employee cynicism and task performance (β = 0.0543), CI [0.0096, 0.0983], supporting hypothesis 5 (see Table 4).

**TABLE 3:** Direct and indirect effects.

Direct effects	β	*S.E.*	*t*	*p*
Role stress → knowledge hiding	0.256	0.058	4.41	0.000
Job insecurity→knowledge hiding	0.271	0.052	4.67	0.000
Employee cynicism→ knowledge hiding	0.345	0.059	5.84	0.000
Knowledge hiding→task performance	–0.053	0.028	–1.89	0.06

**TABLE 4:** Mediation –Bootstraps results.

Path’s effects	B	S.E	LL95%	UL95%
Role stress→knowledge hiding→task performance	0.835	0.0191	0.0472	0.1219
Job insecurity →knowledge hiding → task performance	0.0689	0.0212	0.0264	0.1105
Employee cynicism→knowledge hiding→task performance	0.0543	0.0226	0.0096	0.0983

### Moderation Analysis

**FIGURE 2:**
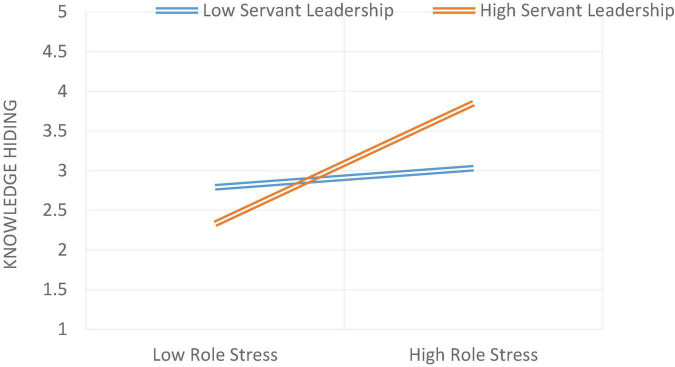
The moderating role of servant leadership between role stress and Knowledge hiding.

**FIGURE 3:**
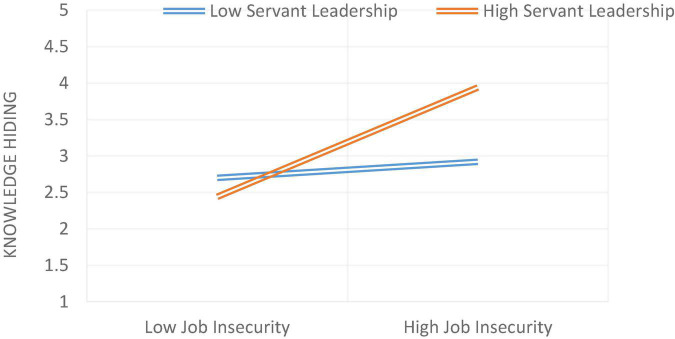
The moderating role of servant leadership between job insecurity and knowledge hiding.

**FIGURE 4:**
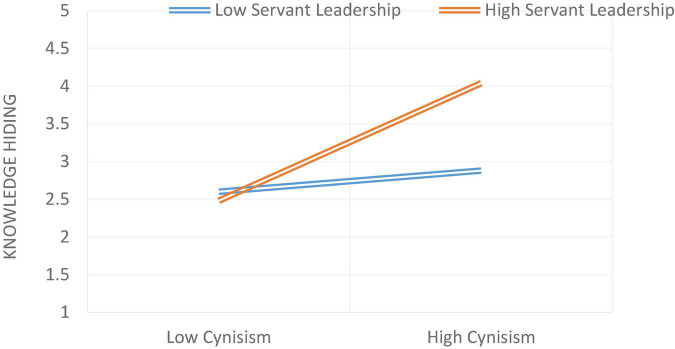
The moderating role of servant leadership between cynicism and knowledge hiding.

Model 1 of Process Macro by Hayes was used to test the moderation hypothesis. On Aiken et al. (1991) recommendation, the moderator and independent variables were mean-centered. Hypothesis 6 was about the moderating effect by stating that servant leadership moderates the relationship between role stress and knowledge hiding. The relationship will be weaker in the case of high servant leadership and stronger in the case of low servant leadership. As stated in Table 5, it was found that the interaction term had a statistically significant effect on knowledge hiding (β = 0.3652***), indicating that servant leadership moderated the relationship between role stress and knowledge hiding. The moderation graph for the relationship between role stress and knowledge hiding is given in Figure 2. As stated in Table 6, it was found that the interaction term had a statistically significant effect on knowledge hiding (β = 0.1910***), indicating that servant leadership moderated the relationship between job insecurity and knowledge hiding (Figure 3). The moderation graph for the relationship between job insecurity and knowledge hiding is given in Figure 2. Further, stated in Table 7, it was found that the interaction term had a statistically significant effect on knowledge hiding (β = 0.1926***), indicating that servant leadership moderated the relationship between employee cynicism and knowledge hiding. The moderation graph for the relationship between job insecurity and knowledge hiding is given in Figure 4.

**TABLE 5:** Moderation analysis.

Variables	Servant leadership (W)					
	
	Role stress (X)		x		Task performance (Y)	
			
	*B*	SE	*T*	P	95%CI	
					
					LL	UL
Constant	6.5011	0.9531	6.8213	0.0000	4.6268	8.3754
Role stress	–0.9899	0.2582	–3.8340	0.0001	–1.4976	–0.4821
Servant leadership	–1.3496	0.2835	–4.7610	0.0000	–1.9071	0.5104
Interaction	0.3652	0.0738	4.9446	0.0000	0.2199	0.5104

**TABLE 6:** Moderation analysis.

	Servant leadership (W)					
	
Variables	Job insecurity (X)		x		Task performance (Y)	
			
	*B*	SE	*t*	P	95% CI	
					
					LL	UL
Constant	4.8047	0.9643	4.9828	0.0000	2.9084	6.7011
Job insecurity	–0.5502	0.2614	–2.1045	0.0360	–1.0644	–0.0361
Servant leadership	–0.6777	0.2682	–2.5269	0.0119	–1.2051	–0.1503
Interaction	0.1910	0.0691	2.7651	0.0060	0.0551	.3268

**TABLE 7:** Moderation analysis.

Variables	Servant leadership (W)					
	
	Employee cynicism (X)		x		Task performance (Y)	
			
	*B*	SE	*t*	P	95% CI	
					
					LL	UL
Constant	4.7739	0.9998	4.7749	0.0000	2.8077	6.7400
Employee cynicism	–0.5903	0.2810	–2.1010	0.0330	–1.1428	–0.0378
Servant leadership	–0.6288	0.2832	–2.2201	0.0270	–1.1858	–0.0718
Interaction	0.1926	0.0754	2.5552	0.0110	0.0444	0.3409

## Discussion

To investigate the impacts of role stress, job insecurity, and cynicism on knowledge hiding behaviour and the effects of knowledge hiding on task performance. According to the study findings, role stress, job insecurity, and cynicism were linked positively with knowledge hiding behaviour. Role stress is often shown in the form of receiving a work assignment for which there are insufficient resources to complete or receiving work requests from two or more people who are incompatible with one another (Khattak et al., 2021). Boz Semerci (2019) explains that employees who feel role stress are more likely to perceive that they lack resources in the workplace, motivating them to maintain their relevant knowledge to prevent their resources from being lost. Job insecurity typically manifests as a fear of losing one’s job or a sense of apprehension about the future of one’s career (Vander Elst et al., 2014; Khan et al., 2022a; Saeed et al., 2022). This perception can make employees feel uncertain about their jobs, leading them to hide knowledge. Employees become cynic when they doubt their value and contribution to the organisation. To avoid being self-serving, cynical employees try to keep the knowledge to themselves. The COR theory fits with these findings (Hobfoll, 1989). When confronted with the potential of resource loss (role stress, job insecurity, and cynicism), employees are more likely to feel psychological pressures, which in turn motivates them knowledge hiding to avoid resource loss (Hobfoll, 1989; Guo et al., 2020). This research, however, adds to the COR theory by highlighting that organisational instability leads to negative attitudes, i.e., role stress, job insecurity and cynicism. These negative attitudes are significant precursors of knowledge hiding.

This study also examined how knowledge hiding affects task performance, consistent with previous findings of (Saeed et al., 2022). Employees who hide their expertise are more likely to engage in less social interactions and information exchanges, resulting in lower productivity at the workplace. Singh (2019) stated that role stress, job insecurity, and cynicism positively affect knowledge hiding, and knowledge hiding has a detrimental impact on task performance (Lei et al., 2019; Khan et al., 2022b). Using COR theory, this study investigated how, when, and why servant leadership might attenuate the effect of negative attitudes that foster culture of knowledge hiding.

### Theoretical Implications

The three main contributions this study adds to the literature. First, this research adds to the body of knowledge by expanding COR theory and examine knowledge hiding and its antecedents in the times of organisational crisis. This study also studied role stress, cynicism and job insecurity as antecedents of knowledge-hiding behaviour. Our study stated that workers are more prone to participate in knowledge hiding behaviour because they are more likely to suffer role stress, job insecurity, and cynicism. Second, this research examines how role stress, job insecurity, and cynicism affect task performance via the mediation role of knowledge hiding behaviour. This research adds to our knowledge of the processes that underlie knowledge hiding and its causes and effects. There has been a previous study on the antecedents’ direct influence on knowledge hiding and knowledge hiding on task performance (Singh, 2019). A significant effort is being made to understand the basic process of knowledge hiding and how it connects its precursors and effects. To minimise additional resource loss, employees may hide knowledge to avoid role stress, job insecurity, and cynicism; this leads to decreased task performance due to the knowledge hiding behaviour. Future studies should look at the mediation function of knowledge hiding to understand better the psychological processes that connect knowledge concealing’s hiding causes and effects.

As a third addition, servant leadership acts as a moderator in the interaction between antecedents and knowledge hiding behaviour. To the best of our knowledge, the function of servant leadership as a moderator in the relationship between knowledge hiding and its antecedents has not yet been investigated. Previous researchers have done considerable studies on servant leadership and task performance, such as Lei et al. (2019). Still, its moderating role in reducing the negative impact of antecedents on knowledge hiding behaviour has gotten little attention in the literature. According to the findings of this study, servant leadership has a positive impact on lowering role stress perceptions, job insecurity and employee cynicism (Khan et al., 2022b). Demonstrating the moderating influence of servant leadership gives insight into the border scenarios in which servant leadership may be used to create a favourable working environment for employees. Workers are more likely to perceive psychological constraints in such an environment, leading to undesirable behaviours such as knowledge hiding. Further research on the moderating effect of servant leadership may be necessary to understand better future methods for reducing knowledge hiding behaviour. Finally, this study combines three linked concepts to form a conceptual model, which is subsequently validated in the field using empirical data. Furthermore, this article contributes to the literature by evaluating the impact of organisational crises in the context of Chinese economy.

### Practical Implications

There are a few practical suggestions derived from the outcomes of this study. First, organisations need to minimise role stress to prompt knowledge-hiding behaviour. For this purpose, managers should consider employees’ capabilities before allocating tasks. According to this study, knowledge hiding behaviour could be reduced by decreasing job insecurity and cynicism as both these directly impart employees task performance. Developing adequate policies for compensating and encouraging employees, offering work enrichment and empowerment, and considering job re-designing by managers can promote sharing resources in a caring and well-established working environment. Leadership must encourage employees and push them toward success in their goals and career as information sharing should be a part of organisational vision and take negative consequences associated with knowledge hiding into consideration for proper development of the organisation. In such cases, employees will be more motivated to share knowledge and reduce knowledge hiding behaviour, proving a better development option for organisations.

### Limitation and Future Research

Some limitations linked with this study offer slots to be considered for the future. First, the conceptual framework of this research is designed for a single developing country, i.e., China only. So, different future models for different developing and developed countries could be designed to enhance this research. This study data were collected from single sources and three different periods to reduce common method bias as guided by Podsakoff et al. (2012). Moreover, different respondents like supervisor-employee dyads could be targetted in the future to expand this study’s span. Lack of personality traits and mindfulness variables are also a limitation associated with this study, so this variable could be added in the future. The current study has only explored servant leadership’s controlling role in the relationship between knowledge hiding and its antecedent. More diverse sets of leadership styles could also be studied in the future. The impact of knowledge hiding behaviour on innovation or productivity could also be investigated in the future, as this study has focussed on just task performance due to knowledge hiding. Apart from these limitations, this study convincingly addressed our research objectives.

## Data Availability Statement

The original contributions presented in this study are included in the article/supplementary material, further inquiries can be directed to the corresponding author.

## Ethics Statement

Ethical review and approval was not required for the study on human participants in accordance with the local legislation and institutional requirements. Written informed consent from the patients/participants or patients/participants legal guardian/next of kin was not required to participate in this study in accordance with the national legislation and the institutional requirements.

## Author Contributions

ZT and CT contributed to the conception and design of the study. CT organised the database. FA and MK performed the statistical analysis. MC, CT, and FA wrote the first draft of the manuscript. MK and MC wrote sections of the manuscript. All authors contributed to manuscript revision, read, and approved the submitted version.

## Conflict of Interest

The authors declare that the research was conducted in the absence of any commercial or financial relationships that could be construed as a potential conflict of interest.

## Publisher’s Note

All claims expressed in this article are solely those of the authors and do not necessarily represent those of their affiliated organizations, or those of the publisher, the editors and the reviewers. Any product that may be evaluated in this article, or claim that may be made by its manufacturer, is not guaranteed or endorsed by the publisher.
